# Artificially induced MAIT cells inhibit *M. bovis* BCG but not *M. tuberculosis* during in vivo pulmonary infection

**DOI:** 10.1038/s41598-020-70615-9

**Published:** 2020-08-12

**Authors:** Huifeng Yu, Amy Yang, Steven Derrick, Jeffrey Y. W. Mak, Ligong Liu, David P. Fairlie, Siobhan Cowley

**Affiliations:** 1 Laboratory of Mucosal Pathogens and Cellular Immunology, Division of Bacterial Parasitic and Allergenic Products, Center for Biologics Evaluation and Research, U.S. Food and Drug Administration, Silver Spring, MD USA; 2 Institute for Molecular Bioscience, The University of Queensland, Brisbane, QLD 4072 Australia; 3 Australian Research Council Centre of Excellence in Advanced Molecular Imaging, The University of Queensland, Brisbane, QLD 4072 Australia

**Keywords:** Immunology, Diseases

## Abstract

There is significant interest in targeting MAIT cells with immunostimulatory agents to enhance immune responses. *Mycobacterium tuberculosis* (*M. tb*.) is a pervasive respiratory disease that could benefit from treatments that augment immunity. Here we investigate the role of MAIT cells in *M. tb*. infection and the potential for MAIT cell-targeted immunotherapy to control bacterial burdens. We find that MAIT cells fail to substantially accumulate in the lungs during murine pulmonary *M. bovis* BCG and *M. tb*. infections but this defect is overcome by intranasal installation of a TLR2/6 agonist and a MAIT cell antigen. Although artificially induced MAIT cells produce important cytokines in both infections, they control BCG but not *M. tb*. growth in the lungs. Correspondingly, *M. tb*.-infected mouse macrophages are relatively resistant to MAIT cell antimicrobial activities in vitro. Thus, MAIT cell antigen-mediated immunotherapy for *M. tb.* presents a complex challenge.

## Introduction

Mucosal associated invariant T (MAIT) cells are a subpopulation of innate-like T cells that express a semi-invariant T cell receptor α chain paired with a variant Vβ chain^1–5^. MAIT cells recognize bacterial metabolites presented by the major histocompatibility complex class I-related (MR1) protein^6,7^. Many studies have demonstrated that MAIT cells respond to a wide variety of pathogens in vitro by rapidly secreting IFN-γ, TNF, IL-17, and Granzyme B^8–13^. However, a non-redundant role for MAIT cells has only been demonstrated for a few pathogens during in vivo infection, including the live attenuated vaccines *Francisella tularensis* live vaccine strain (LVS) and *Mycobacterium bovis* Bacillus Calmette Guerin (BCG)^9,14,15^. BCG is the only vaccine available for prevention of *M. tb.* infection, although it exhibits highly variable protective efficacy (0–80%) against adult pulmonary tuberculosis^16,17^. Since one third of the world’s population is estimated to be infected with *M. tb.*, a better vaccine and new therapies are urgently needed to combat this disease. In this regard, the innate antibacterial activities of MAIT cells may serve as a useful therapeutic target, although the role of MAIT cells in defense against *M. tb*. remains to be fully defined.

Previous studies have reported that MAIT cells protect against non-tuberculosis mycobacteria and BCG infections in mice^12,18,19^. However, recent studies detected few MAIT cells in the airways of macaques during *M. tb* infection^20,21^, and MAIT cell deficiencies were observed in the pleural effusions of patients with active tuberculosis^22^. Collectively, these data suggest that MAIT cells may not be abundant in *M. tb* infection. Since intranasal administration of the MAIT cell antigen 5-OP-RU and a TLR agonist strongly induced MAIT cell accumulation in the lungs of naïve mice^23^, a similar treatment regimen might promote MAIT cell responses to control *M. tb*. infection. Using murine pulmonary infection models, here we find that MAIT cells fail to substantially accumulate in the lungs of mice during BCG and *M. tb.* infection, exhibiting 100-fold fewer MAIT cells as compared to *F. tularensis* LVS infection. Intranasal treatment with a TLR2/6 agonist and 5-OP-RU greatly enriches MAIT cell numbers in the lungs of BCG and *M. tb*.-infected mice, and these “induced” MAIT cells produce critical cytokines. Surprisingly, although artificially induced MAIT cells inhibit BCG growth in the lungs of infected mice, they do not affect *M. tb* growth. Our data indicate that *M. tb* evades the action of MAIT cell antimicrobial activities.

## Results and discussion

### MAIT cells do not accumulate in the lungs during BCG intranasal infection

**Figure 1:**
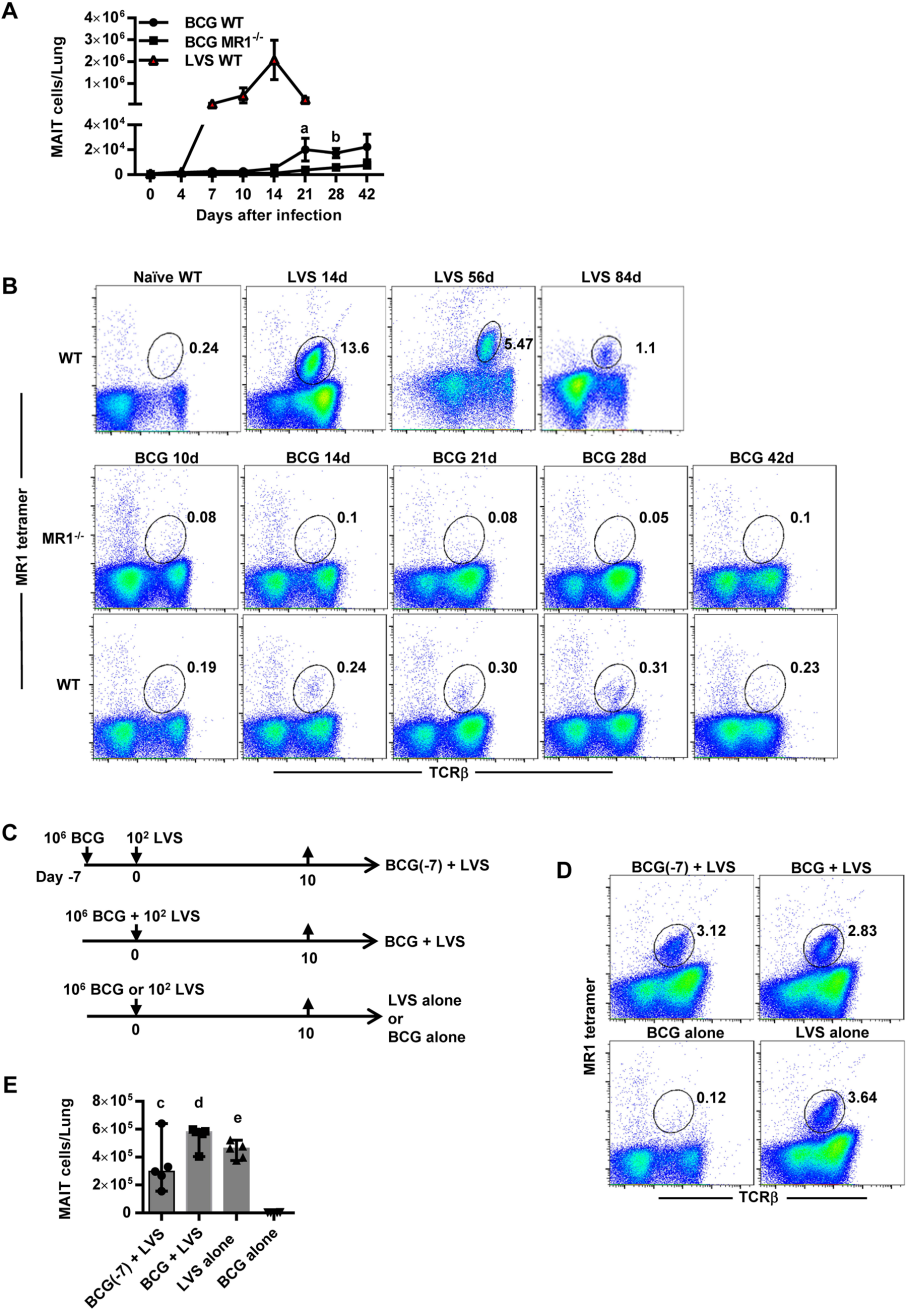
MAIT cells exhibit limited accumulation in the lungs during murine BCG intranasal infection. (**A**) The total number of MR1-5-OP-RU tetramer^+^ MAIT cells in the lungs of WT and MR1^−/−^ mice infected IN with BCG, and WT mice infected IN with LVS. a = *P* = 0.02 and b = *P* = 0.01 as compared to MR1^−/−^ mice. Data show the mean ± SEM (n = 5 mice). (**B**) Flow cytometry plots showing the percentage of MAIT cells in total lung cells of WT mice during LVS IN infection, and WT and MR1^−/−^ mice during BCG IN infection at the indicted time points. (**C**) Schematic representation of the LVS and BCG co-infection experiment. WT mice were administrated BCG IN alone, LVS IN alone, BCG plus LVS IN simultaneously, or BCG IN followed by LVS IN seven days later. Lung cells were collected on day 10. (**D**) Flow cytometry plots showing the percentage of MAIT cells in total lung cells of mice on day 10 after the treatments depicted in (**C**). (**E**) The number of MR1-5-OP-RU tetramer^+^ MAIT cells in the lungs of mice on day 10 after the treatments depicted in (**C**). c = *P* = 0.009, d and e = *P* < 0.0001 as compared to BCG alone mice. Data show individual values, median, and the range (n = 4–5 mice). Data are representative of 3 independent experiments.

Since MAIT cells accumulate to high levels in the lungs of mice during pulmonary LVS infection^9^, we sought to determine whether MAIT cells respond similarly to intranasal (IN) BCG infection. LVS generates an acute pulmonary infection in mice with a peak in bacterial growth at day 7 and clearance by approximately day 18^9^. In contrast, BCG IN infection peaks by approximately day 21 and takes more than two months to clear. After LVS IN infection, MAIT cells in the lungs of WT mice significantly increased as early as 7 days after infection and peaked on day 14 (~ 2 × 10^6^ ± 5 × 10^5^ MAIT cells/lung, as compared to 775 ± 62 MAIT cells/lung in naïve mice, *P* < 0.0001) (Fig. 1A). Significantly elevated numbers of MAIT cells persisted in the lungs of LVS-infected mice for months, with MAIT cells constituting 3.5% ± 0.2% and 1.1% ± 0.02% of total lung cells on days 56 and day 84 after infection, respectively (as compared to 0.2% ± 0.04% of total lung cells in naïve mice, *P* < 0.0001, Fig. 1B). In contrast, after BCG IN infection, MAIT cell numbers peaked in the lungs on approximately day 21, with an average number of 2 × 10^4^ ± 4.5 × 10^3^ MAIT cells/lung, approximately 100 times less than LVS-infected mice on day 14. Moreover, MAIT cells in the lungs of BCG-infected mice remained low on days 28, 42, and 140 (Fig. 1A, and data not shown), although they were significantly higher than that observed in MR1^−/−^ mice, which lack MAIT cells. Overall, MAIT cells did not exceed 0.31% of total lung cells at any of the time points tested in BCG-infected mice, whereas they peaked at 13.6% of total lung cells in LVS-infected mice (Fig. 1B). Of note, the number of conventional TCRβ^+^ T cells (CD4^+^ T cells, CD8^+^ T cells) in the lungs of BCG-infected mice was only threefold less than LVS-infected mice (Suppl. Fig. 1A,B). These data show that MAIT cell accumulation in the lungs of BCG-infected mice is impaired as compared to LVS infection, and that this phenomenon is more severe for MAIT cells than conventional TCRβ^+^ T cells. Since rapid MAIT cell responses depend on MR1 antigen presentation and secondary signals that can be provided by TLR ligation^23^, the inability of BCG to stimulate robust MAIT cell expansion could stem from relatively poor antigen presentation, production of inhibitory MAIT cell antigens^7^, and/or inadequate TLR responses.

To gain insight into whether BCG actively impairs MAIT cell accumulation or whether LVS activates an immunostimulatory program that BCG lacks, we co-infected mice with LVS and BCG. WT mice were inoculated IN with (1) BCG 7 days prior to LVS IN infection (BCG(− 7) + LVS), (2) BCG and LVS (BCG + LVS) simultaneously, (3) LVS alone, or (4) BCG alone (Fig. 1C), and MAIT cell numbers in the lungs were evaluated 10 days later. As shown in Fig. 1D,E, mice infected with BCG alone had extremely low numbers of MAIT cells in their lungs, whereas those infected with LVS alone exhibited significant MAIT cell accumulation. Mice administered both BCG and LVS exhibited a significant increase in MAIT cells as compared to mice infected with BCG alone (*P* < 0.0001) and were not significantly different than mice inoculated with LVS alone. MAIT cell numbers increased regardless of whether BCG inoculation occurred a week prior to, or at the same time as, LVS infection. Overall, these data show that co-infection with LVS overcomes the impaired accumulation of MAIT cells in BCG infection, indicating that BCG does not irrevocably suppress MAIT cells but instead fails to activate signals that lead to their accumulation.

### Effect of a TLR2/6 agonist and MAIT cell antigen on MAIT cell accumulation and inhibition of BCG growth

**Figure 2:**
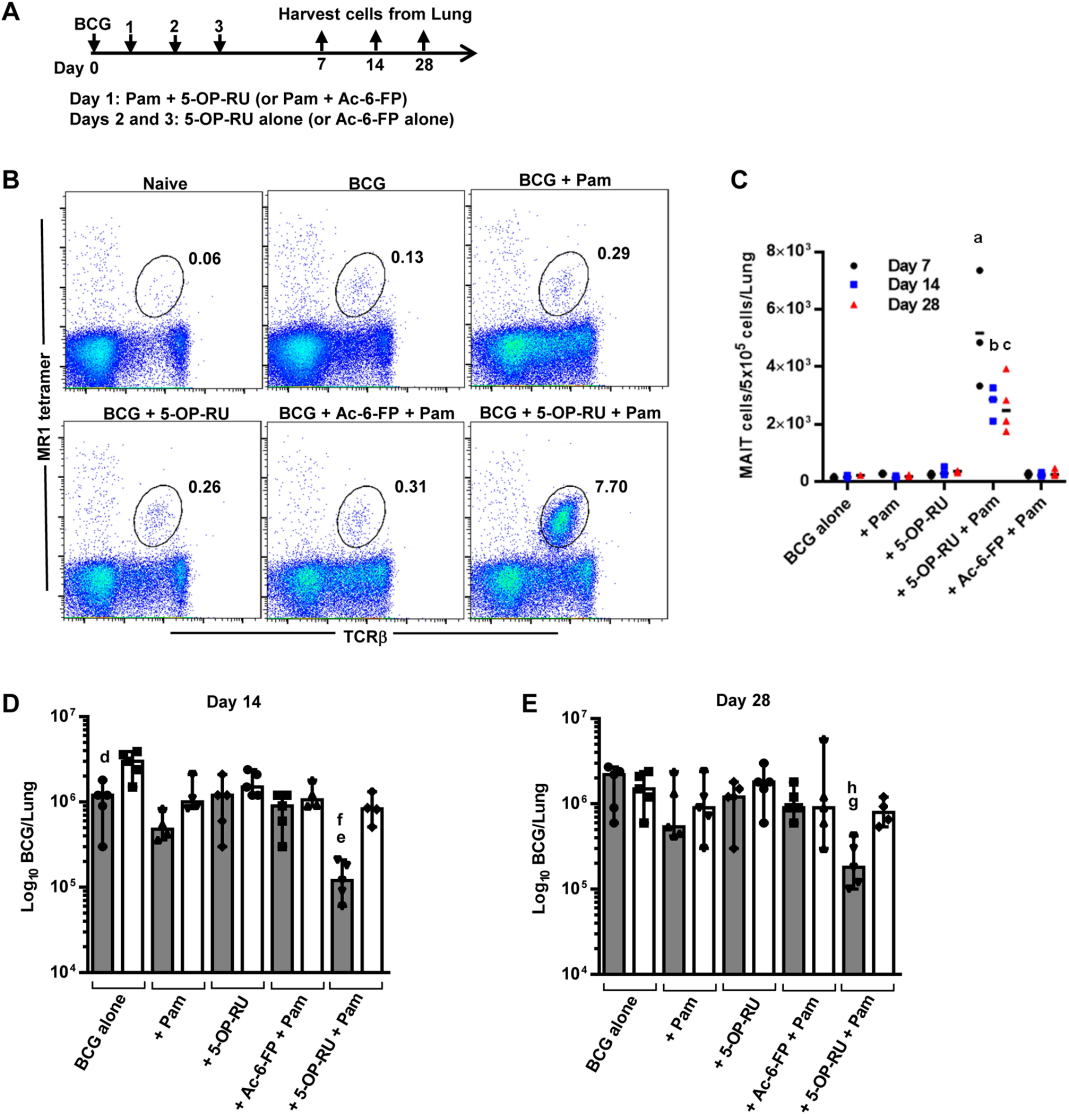
Intranasal instillation of Pam_2_CSK_4_ plus 5-OP-RU induces MAIT cell accumulation and control of BCG growth in the lungs. (**A**) Schematic representation of the MAIT cell induction experiment. Mice were intranasally administered BCG on day 0, MAIT cell ligand 5-OP-RU (or Ac-6-FP as negative control) + Pam on day 1, and 5-OP-RU or Ac-6-FP alone on days 2 and 3. Lung cells were assessed by flow cytometry on days 7, 14, and 28. a, b, and c = *P* < 0.0001 as compared to BCG + Pam WT mice (n = 5 mice). (**B**) Flow cytometry plots showing the percentage of MAIT cells in total lung cells of mice on day 7 after the treatments depicted in (**A**). (**C**) The number of MR1-5-OP-RU tetramer^+^ MAIT cells in the lungs of mice on days 7, 14, and 28 after the treatments depicted in (**A**). Data show individual values and the median (n = 3 mice). BCG CFUs in the lungs of mice following the treatments depicted in (**A**) on day 14 (**D**) and day 28 (**E**) after infection (WT mice = grey bars, MR1^−/−^ mice = white bars). d = *P* = 0.06, e = *P* = 0.01, and g = *P* = 0.004 as compared to similarly treated MR1^−/−^ mice. f and h = *P* = 0.02 as compared to BCG + Pam WT mice. Bar graphs show individual values, median, and the range (n = 5 mice). Data are representative of 3 independent experiments.

Previous studies have shown that IN administration of a TLR2 agonist and the MAIT cell activating antigen 5-OP-RU increased MAIT cell numbers in the lungs of naïve mice^23^. Therefore, we tested the ability of the TLR2/6 agonist Pam_2_CSK_4_ (Pam) and 5-OP-RU to promote MAIT cell accumulation in BCG-infected mice. WT mice were IN administered BCG on day 0, Pam + 5-OP-RU on day 1, then 5-OP-RU alone on days 2 and 3, and MAIT cell frequencies in the lungs were assessed on days 7, 14 and 28 (Fig. 2A). The ligand Ac-6-FP, which binds MR1 but inhibits MAIT cell activation, was employed as a control. As shown in Fig. 2B,C, MAIT cell numbers significantly increased in BCG-infected mice that received Pam + 5-OP-RU as compared to control mice. Importantly, although MAIT cells were most abundant on day 7, MAIT cell numbers remained significantly elevated for at least a month after the infection (Fig. 2C). In contrast, the numbers of CD4^+^ T cells and CD8^+^ T cells among the different treatment groups did not show significant differences at any of the indicated time points (Suppl. Fig. 1C,D).

To determine whether these “induced” MAIT cells can control BCG infection, bacterial growth in the lungs of WT and MR1^−/−^ mice was assessed on day 14 (Fig. 2D) and day 28 (Fig. 2E) after BCG IN infection. WT mice treated with Pam + 5-OP-RU had significantly lower bacterial CFUs in their lungs at both time points as compared to similarly treated MR1^−/−^ mice, which lack MAIT cells. Importantly, this protective effect was only observed in WT mice given Pam + 5-OP-RU, and none of the control treatments. Thus, exposure of mice to a MAIT cell activating antigen and a TLR agonist significantly reduces BCG growth in a MAIT cell-dependent manner that is sustained for at least a month after infection.

### Effect of a TLR2/6 agonist and MAIT cell antigen on MAIT cell accumulation and inhibition of *M. tb*. growth

**Figure 3:**
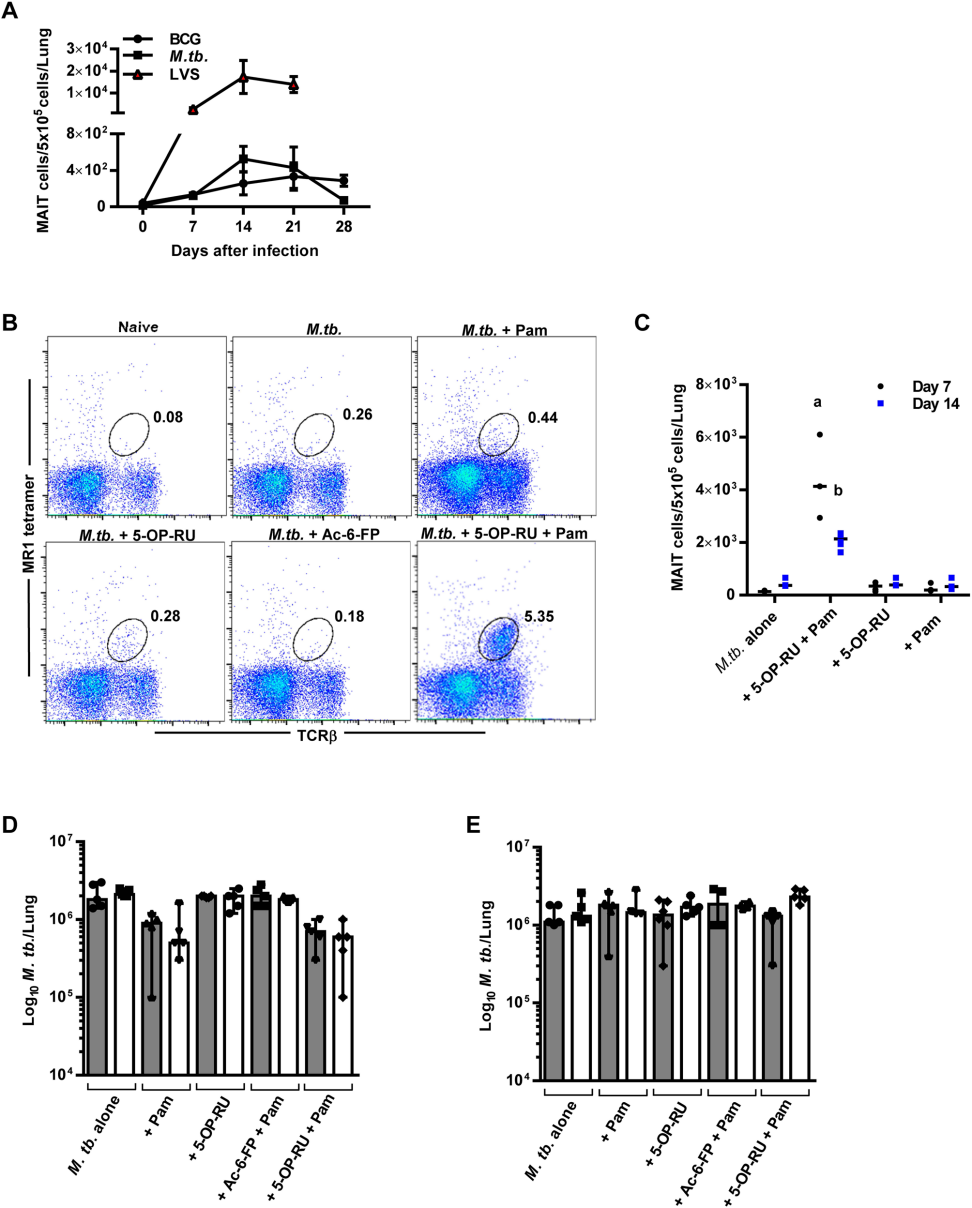
Intranasal instillation of Pam_2_CSK_4_ plus 5-OP-RU induces MAIT cell accumulation but not control of *M. tb.* growth in the lungs. Mice were intranasally administered 5-OP-RU + Pam according to the same schedule as Fig. 2A. (**A**) The number of MR1-5-OP-RU tetramer^+^ MAIT cells in the lungs of WT mice infected with BCG IN, LVS IN, or *M. tb*. via aerosol. Data show the mean ± SEM (n = 5 mice). (**B**) Mice were administered *M. tb*. aerosol on day 0, MAIT cell ligand 5-OP-RU + Pam (or Ac-6-FP + Pam as negative control) IN on day 1, and 5-OP-RU or Pam alone IN on days 2 and 3. Flow cytometry plots show the percentage of MAIT cells in total lung cells on day 7. (**C**) The number of MR1-5-OP-RU tetramer^+^ MAIT cells in the lungs on days 7 and 14 after the treatments described in (**B**). Data show individual values and the median (n = 3 mice) (**D**) *M. tb.* CFUs in the lungs on day 14 following the treatments described in (**A**) (WT mice = grey bars, MR1^−/−^ mice = white bars). (**E**) After 29 days of *M. tb.* aerosol infection, mice were treated IN with Pam + 5-OP-RU, followed by two IN doses of 5-OP-RU on days 30 and 31. The graph depicts *M. tb.* lung CFUs on day 44 after *M. tb.* aerosol infection (WT mice = grey bars, MR1^−/−^ mice = white bars). a and b = *P* < 0.0001 as compared to *M. tb*. + Pam WT mice. Bar graphs show individual values, the median, and the range (n = 5 mice) and are representative of 2 independent experiments.

We next sought to determine whether induced MAIT cells could function as a therapy to reduce the *M. tb.* bacterial burden in the lungs. WT mice given a low dose *M. tb* aerosol infection failed to accumulate large numbers of MAIT cells in their lungs (Fig. 3A,B) and harbored approximately 100-fold fewer MAIT cells than observed during LVS IN infection. Additionally, the numbers of conventional TCRβ^+^ T cells (CD4^+^ T cells, CD8^+^ T cells) present in the lungs during *M. tb.* infection exhibited a threefold reduction as compared to LVS infection (Suppl. Fig. 2A,B). Next, WT mice given an aerosol *M. tb.* infection were treated with Pam + 5-OP-RU according to the same schedule described in Fig. 2A, and MAIT cell frequencies in the lungs were assessed on days 7 and 14. As above, inhibitory ligand Ac-6-FP was used as a control. As shown in Fig. 3B,C MAIT cell numbers were significantly augmented in the lungs of *M. tb.-*infected mice treated with Pam + 5-OP-RU as compared to control mice on days 7 and 14 after infection. This data demonstrates that, similar to BCG infection, exposure to a MAIT cell activating antigen and a TLR agonist can induce MAIT cell accumulation in *M. tb-*infected lungs.

Next, WT and MR1^−/−^ mice were treated with Pam + 5-OP-RU according to the schedule outlined in Fig. 2A, and *M. tb*. growth in the lungs was assessed on day 14 after aerosol infection, when the greatest growth suppression was observed in our BCG studies (Fig. 3D). Surprisingly, unlike our results for BCG infection, *M. tb.* growth in the lungs of WT and MR1^−/−^ mice inoculated with Pam + 5-OP-RU were not significantly different, despite the high number of MAIT cells detected in the lungs of WT mice (Fig. 3B). We next investigated the possibility that induced MAIT cells could provide a protective effect during the chronic phase of infection. Mice were inoculated IN with Pam + 5-OP-RU 29 days after a low dose *M. tb* aerosol infection, followed by two doses of 5-OP-RU on days 30 and 31. The *M. tb* bacterial burdens in the lungs showed no significant differences between any of the groups on day 14 after treatment (44 days after *M. tb.* infection) (Fig. 3E) despite a MAIT cell population of 3.6 ± 0.2% in the lungs of mice given Pam + 5-OP-RU (*P* < 0.0001 as compared to infected mice given Pam, data not shown). Thus, MAIT cells induced during aerosol *M. tb* infection are incapable of reducing the bacterial load in the lungs in both the early and chronic stages of infection. This is in contrast to our findings with BCG-infected mice as well as studies in the murine model of *Legionella longbeacheae* infection, where the forced expansion of MAIT cells reduced bacterial growth in the lungs^15^.

**Figure 4:**
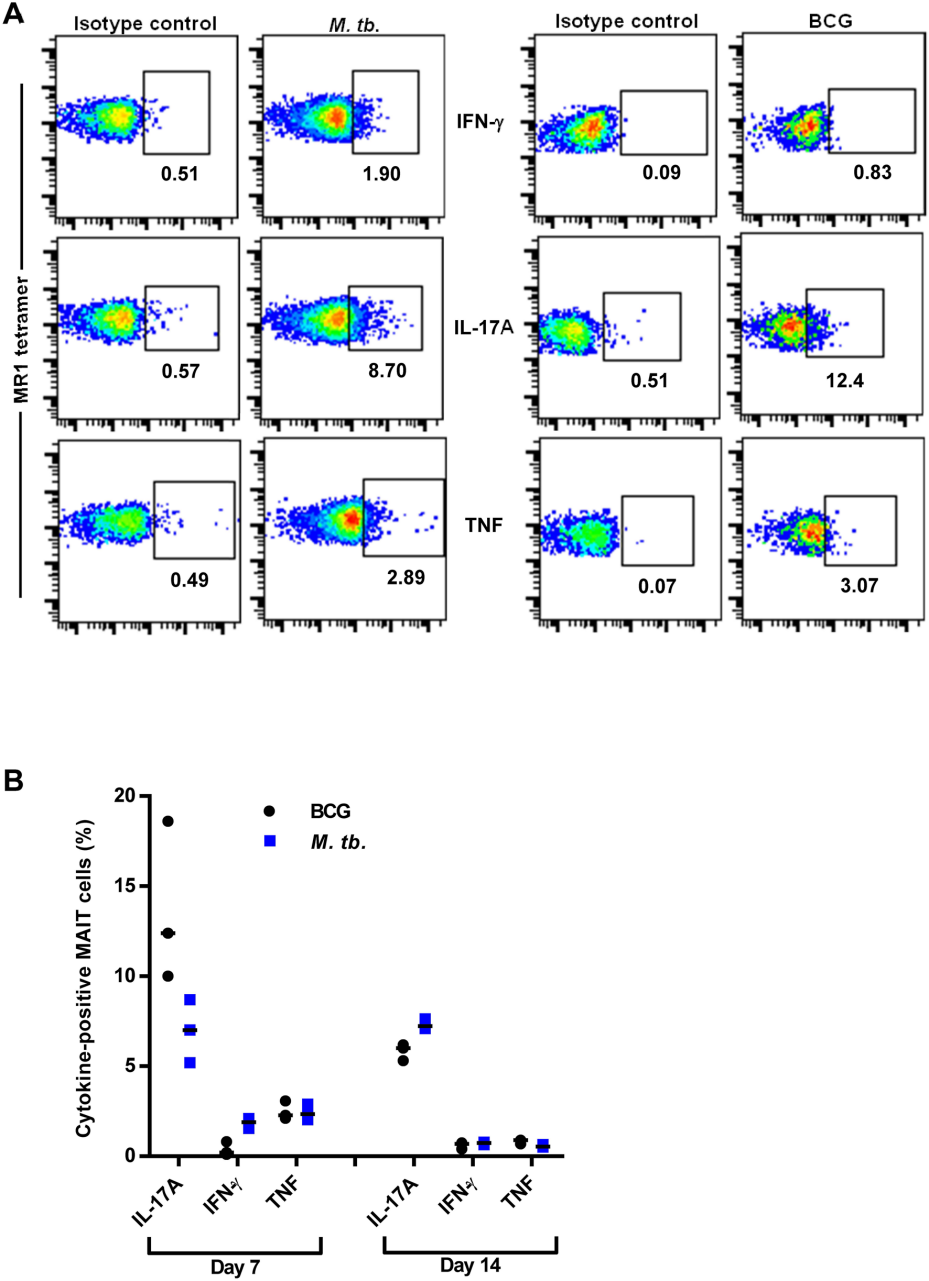
Induced MAIT cells produce cytokines during both BCG and *M. tb.* pulmonary infection. (**A**) Flow cytometry dot plots of IFN-γ, TNF, and IL-17A production by MR1-5-OP-RU tetramer^+^ MAIT cells in the lungs of mice after BCG IN or *M. tb*. aerosol infections and IN instillation of Pam + 5-OP-RU according to the schedule depicted in Fig. 2A. Cytokine production was assessed on day 7 after infection. Cells were first gated on live singlet MR1-5-OP-RU tetramer^+^ MAIT cells. (**B**) The percentage of cytokine-positive MAIT cells present in the lungs on days 7 and 14 after infection with BCG IN or *M. tb*. aerosol and Pam + 5-OP-RU treatment. Data show individual values and the median and are representative of 2 independent experiments (n = 3 mice per experiment).

Next, we investigated the possibility that the MAIT cell population induced during *M. tb.* infection is functionally inert. To this end, we compared the cytokine production of the induced MAIT cells harvested from the BCG and *M. tb* pulmonary infection experiments (Figs. 2, 3). Consistent with MAIT cells in other pulmonary infection models^23,24^, induced MAIT cells in the lungs of BCG-infected mice produced high levels of IL-17A, and very low but detectable levels of IFN-γ and TNF on day 7 after infection (Fig. 4A,B). Surprisingly, induced MAIT cells present in the lungs of *M. tb-*infected mice exhibited a similar pattern of cytokine production and were not significantly different from BCG-infected mice (Fig. 4A,B). In both infections, MAIT cell cytokine production was highest at day 7 and sustained through day 14. Consistent with our finding that *M. tb.* does not suppress MAIT cell activities, MAIT cells in the peripheral blood of macaques infected with *M. tb*. exhibited increased frequencies of Ki67 expression, indicating activation^25^. Further, MAIT cells in pleural effusions from *M. tb*.-infected individuals expressed IFN-γ and IL-17A upon restimulation in vitro^26,27^. Overall, our data show that functionally competent MAIT cells can be artificially induced to accumulate in the lungs of *M. tb.-*infected mice, but these cells lack the capacity to inhibit *M. tb.* growth in vivo.

### MAIT cells strongly inhibit BCG growth but not *M. tb*. growth in BMMΦ in vitro

**Figure 5:**
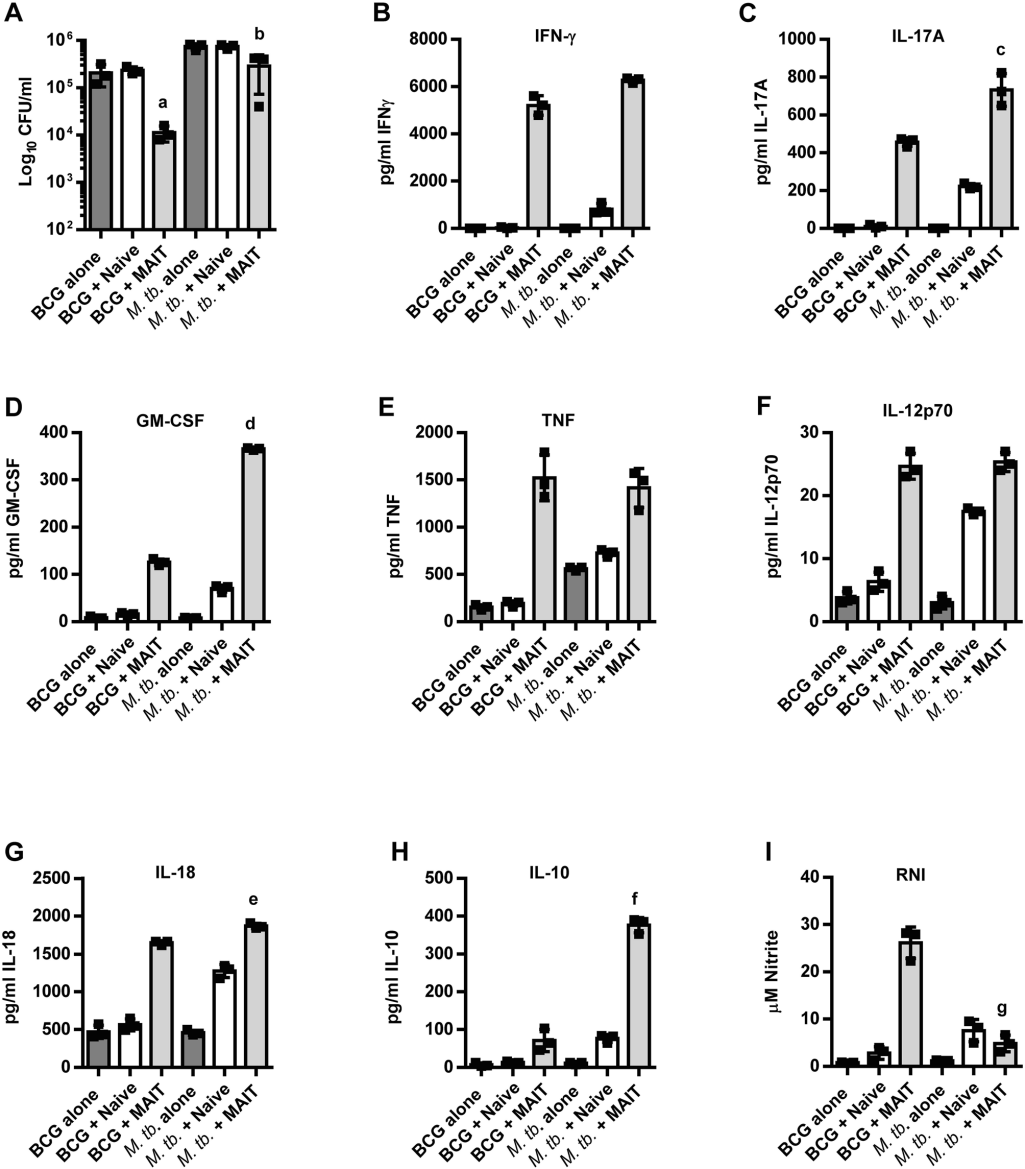
MAIT cells inhibit BCG growth but poorly control *M. tb.* growth in macrophages. WT BMMΦ were infected with BCG or *M. tb.* and cultured with MR1-5-OP-RU tetramer^+^ MAIT cells purified from naive Vα19iTg-MR1^+/+^ mice (“+ MAIT”). Naïve WT splenic T cells were used as a negative control (“+ Naïve”). Bacterial growth in the BMMΦs was enumerated on day 7 of co-culture (**A**) a = *P* = 0.007 and b = *P* = 0.027 as compared to “+ Naïve”. Supernatants were collected on day 6 of co-culture and assessed for IFN-γ (**B**), IL-17A (**C**), GM-CSF (**D**), TNF (**E**), IL-12p70 (**F**), IL-18 (**G**), IL-10 (**H**), and RNI (**I**). c = *P* = 0.006, d = *P* < 0.0001, e = *P* = 0.001, f = *P* = 0.0001, and g = *P* = 0.0006 as compared to BCG + MAIT cells. Data are representative of 2 independent experiments and show individual values, the median, and the range from triplicate wells.

To further understand the ability of MAIT cells to control BCG growth but not *M. tb.* growth, MAIT cells were isolated from Vα19iTg MR1^+/+^ mice and co-cultured with BCG or *M. tb.*-infected bone marrow-derived macrophages (BMMΦ) in vitro. Infected BMMΦ co-cultured with naïve WT Thy1.2^+^ cells served as the negative control. Similar to our observations in vivo, in vitro cultured MAIT cells significantly inhibited BCG growth (25-fold as compared to control cell cultures; *P* = 0.007) but only showed a 2.5-fold reduction of *M. tb.* growth as compared to control cell cultures (*P* = 0.027) (Fig. 5A). Previous studies have shown that MAIT cells control BCG intramacrophage growth in vitro in an IFN-γ-dependent, IL-12p40-dependent, and MR1-independent manner^18,19,28^. We similarly found that MAIT cell control of BCG growth was reversed by addition of anti-IFN-γ blocking Abs (data not shown). As shown in Fig. 5B, the levels of IFN-γ in the *M. tb*. cultures were not significantly different from the BCG cultures. In contrast, the levels of IL-17A and GM-CSF were significantly higher in the *M. tb*. cultures (Fig. 5C,D), while TNF was not significantly different (Fig. 5E). Since IFN-γ and GM-CSF mediated MAIT cell antimicrobial activities during in vivo* L. longbeachae* infection^15^, and these two cytokines have been shown to control *M. tb*. intramacrophage growth^29^, it appears that the MAIT cells in our *M. tb*. cultures possess the tools necessary to limit *M. tb*. growth. Further, IL-12p70 and IL-18 were not significantly lower in the *M. tb* co-cultures as compared to the BCG co-cultures (Fig. 5F,G), while IL-2 was similar in both co-cultures (8.1 ± 0.29 pg/ml for BCG and 8.3 ± 0.60 for *M. tb*.; *P* = 0.66). Importantly, IL-12p70 and IL-18 are critical for MAIT cell co-stimulation and activation^30,31^, while IL-2 has been implicated in enhanced MAIT cell responses^32^. The similar quantities of IL-2, IL-12p70, and IL-18 observed in the *M. tb*. and BCG co-cultures are consistent with their comparable levels of MAIT cell IFN-γ production, suggesting that differences in these mediators are not responsible for the poor *M. tb*. growth control. Of note, the Vα19iTg MR1^+/+^ mice used in our in vitro experiments are useful for obtaining large numbers of purified MAIT cells because naïve conventional WT mice contain few MAIT cells^33^. However, because Vα19iTg MR1^+/+^ mice exclusively express the canonical TRAV1-TRAJ33 TCRα gene, MAIT cells from these mice exhibit some notable differences from those found in WT mice, including a significant population of CD4^+^ MAIT cells^12,34^. Although the importance of any differences between transgenic and WT MAIT cells remains unknown, it is apparent that the data from our in vitro experiments reflect our in vivo observations, including the ability of MAIT cells to produce comparable levels of IFN-γ and IL-17A in response to both pathogens and to potently control BCG but not *M. tb* growth. Thus, both in vitro and in vivo, MAIT cells are activated to produce effector cytokines but lack the ability to limit *M. tb*. growth.

### IL-10 is abundant in MAIT cell co-cultures containing macrophages infected with *M. tb.* but not BCG

Since the key cytokines that signify MAIT cell activation and effector activity were abundant in the *M. tb* co-cultures, we next investigated the possibility that inhibitory cytokines and impaired macrophage effector mechanisms may be responsible for the poor control of *M. tb.* infection. We found that MAIT cell co-cultures containing *M. tb.-*infected BMMΦ exhibited significantly higher levels of IL-10 (Fig. 5H) than the BCG co-cultures (*P* = 0.0001), and significantly lower levels of reactive nitrogen intermediates (RNI) (*P* = 0.0006; Fig. 5I). Notably, RNI are key effector molecules generated by macrophages that limit mycobacterial growth. In contrast, IL-10 is an anti-inflammatory cytokine that is detrimental to the outcome of mycobacterial infections in mice and humans^35–37^. Interestingly, IL-10 has direct effects on macrophage antimicrobial activities, including promoting an alternatively activated phenotype, blocking phagosome maturation, and reducing RNI production^38–40^. Our finding that macrophages in *M. tb*. cultures produced limited RNI is consistent with a possible role for IL-10 in impaired macrophage function.

There is significant interest in using MAIT cell antigens as adjuvants or immunotherapeutics to enhance immune responses. In mice and humans, *M. tb.* is a persistent infection that is resistant to eradication by the immune system and is an attractive candidate for immunotherapies that enhance antimicrobial activity. Here we show that MAIT cell accumulation in the lungs during pulmonary BCG and *M. tb*. infection is impaired but can be overcome by intranasal administration of a TLR2/6 agonist and a MAIT cell antigen. However, in contrast to BCG, *M. tb*. was relatively resistant to the antimicrobial activities of MAIT cells both in vitro and in vivo. These data show that targeting MAIT cells as a treatment for *M. tb*. infection poses some unexpected challenges. Our findings pave the way for future studies that explore the impact of IL-10 and other anti-inflammatory cytokines on MAIT cell therapies and provide a greater understating of the impediments to MAIT cell-mediated *M. tb*. interventions.

## Methods

### Bacterial infections, mice, preparation of synthetic ligands, and in vivo MAIT cell induction

*F. tularensis* LVS (ATCC) was grown, frozen, and quantified as previously described^41^. *M. bovis* BCG (Pasteur strain) and *M. tb*. Erdman were grown, frozen and quantified as previously described^41^. All *F. tularensis* LVS and *M. bovis* BCG experiments were performed in a BSL-2 laboratory, while all *M. tb*. experiments were performed in a BSL-3 containment laboratory. Male and female C57BL/6J mice were purchased from The Jackson Laboratory. MR1 KO mice^42^ and Vα19iTgCα^−/−^MR1^+/+^ transgenic mice (which exclusively express the canonical TCR Vα19-Jα33 of murine MAIT cells)^34^ were obtained from Ted Hansen (Washington University in St. Louis, St. Louis, MO) and bred at CBER/FDA. Animals were housed in a barrier environment at CBER/FDA, and all experimental protocols were approved by the FDA White Oak Animal Care and Use Committee. All animal experiments were performed in accordance with the relevant guidelines and regulations. Bacteria were diluted in PBS (Gibco) and IN infections were performed by delivering a total of 1–2 × 10^2^ LVS colony-forming units (CFU) or 10^6^ BCG CFU in a volume of 25 µl to anesthetized mice. *M. tb.* was delivered by aerosol at a concentration that deposits ~ 100 CFU in the lungs over a 30-min exposure in a Glas Col aerosol exposure chamber (Terre Haute, IN)^43^. Synthetic 5-OP-RU (as a DMSO solution) and Ac-6-FP were prepared according to previously published procedures^44,45^. For MAIT cell induction in vivo, the first dose consisted of a combination of 30 μg Pam_2_CSK_4_ (Invivogen) and MAIT cell ligands (5-OP-RU or Ac-6-FP; 25 μl of 12 μM per mouse) delivered IN. The second and third doses consisted of 5-OP-RU or Ac-6-FP (25 μl of 6 μM per mouse) delivered IN.

### Infection of BMMΦ and co-culture with MAIT cells in vitro

BMMΦ were used as antigen-presenting cells and in vitro co-cultured with MAIT cells according to previous publications^9,11,41^. BMMΦ were infected with *M. tb.* or BCG at a multiplicity of infection of 1:100 (bacterium-to-BMMΦ ratio). Vα19iTgCα^−/−^MR1^+/+^ mice were used as the source of MAIT cells for co-culture with infected BMMΦs at a ratio of 1 MAIT cell to 2 BMMΦ. MAIT cells were purified from Vα19iTgCα^−/−^MR1^+/+^ mouse spleens using a PE-labelled 5-OP-RU-MR1 tetramer and anti-PE-beads (Life Technologies) according to the manufacturer's recommendations. Of note, it has previously been shown that the control strain for the Vα19iTgCα^−/−^MR1^+/+^ mice that lack MR1 (Vα19iTgCα^−/−^MR1^−/−^ mice) possesses a large population of MR1 tetramer-reactive T cells and it was concluded that this strain does not serve as a useful control in experiments^12^. For this reason, naïve WT T cells were purified using Life Technologies anti-Thy1.2 beads and used as a non-immune T cell control. The culture supernatants were collected for cytokine analyses (d6) and the BMMΦ were lysed with sterile distilled H_2_O for CFU determinations (d7).

### Flow cytometry and intracellular cytokine staining

The lungs were excised, transferred to a Petri dish, and chopped with sharp scissors until no large pieces were visible, then incubated with 3 ml of DMEM containing 10% FBS, collagenase D (Sigma, 0.5 mg/ml) for 1 h at 37 °C in 5% CO_2_. The cells were filtered through a 40-µm filter, subjected to ACK lysis, and passed through a 40-µm filter again. Live cells were enumerated on a hemocytometer after dilution in Trypan blue. Cells were stained for a panel of murine cell surface markers and analyzed using a BD LSR Fortessa flow cytometer and FlowJo software. Ab clones used included MP6-XT22 (anti-TNF), (anti-IL17A), XMG-6 (anti–IFN-γ), HMb1-1 (anti-CD4), 53-6.7 (anti-CD8α), H57-597 (anti-TCR β-chain) (BioLegend). 5-OP-RU-loaded MR1 MAIT cell tetramers were obtained from the NIH Tetramer Core Facility (Atlanta, GA). Live/Dead Near IR stain (Molecular Probes) was included in all staining protocols. Cells were first gated on singlets and live cells before further analyses. To monitor the expression of TNF, IFN-γ, and IL-17A, lung cells were incubated in cDMEM containing 5 μg/ml Brefeldin A at 37 °C in 5% CO_2_ for 4 h and stained for cell surface markers. Intracellular staining was performed using the BD Biosciences buffer system according to the manufacturer’s instructions. Note that due to limitations in procedures that may be safely used in the BSL-3 laboratory, lung cell numbers for flow cytometry are depicted as MAIT cells/5 × 10^5^ cells/lung and not total MAIT cells/lung.

### Quantitation of cytokines and RNI

Culture supernatants and lung homogenates were assayed by Luminex assay for Cytokine and chemokine 26-Plex Mouse ProcartaPlex Panel 1 (ThermoFisher) according to the manufacturer’s instructions. RNI were quantified using a Griess reagent kit according to the manufacturer’s instructions (ThermoFisher).

### Statistical analyses

All experiments were performed and repeated to assess reproducibility using three to five mice per experimental group unless otherwise stated. Data were analyzed via multiple t tests or one-way ANOVA followed by the Student–Newman–Keuls multiple stepwise comparison (for experiments with more than two experimental groups). A *P* value < 0.05 was considered a significant difference.

## Supplementary information

Supplementary Figures.
