# Supplementary material for: Artificially induced MAIT cells inhibit M. bovis BCG but not M. tuberculosis during in vivo pulmonary infection
Source: Sci Rep. 2020 Aug 12;10:13579. doi: 10.1038/s41598-020-70615-9 (PMC7423888; doi:10.1038/s41598-020-70615-9)
Supplement: Supplementary file 1 — Supplementary Figures. [file 41598_2020_70615_MOESM1_ESM.pdf]

Artificially induced MAIT cells inhibit *M. bovis* BCG but not *M. tuberculosis* during *in vivo* pulmonary infection

Huifeng Yu[1], Amy Yang[1], Steven Derrick[1], Jeffrey Y.W. Mak[2,3], Ligong Liu[2,3], David P. Fairlie[2,3], and Siobhan Cowley[1*]

[1]Laboratory of Mucosal Pathogens and Cellular Immunology, Division of Bacterial Parasitic and Allergenic Products, Center for Biologics Evaluation and Research, U.S. Food and Drug Administration, Silver Spring, Maryland, USA

[2] Institute for Molecular Bioscience, The University of Queensland, Brisbane, Queensland 4072, Australia

[3]Australian Research Council Centre of Excellence in Advanced Molecular Imaging, The University of Queensland, Brisbane, Queensland 4072, Australia

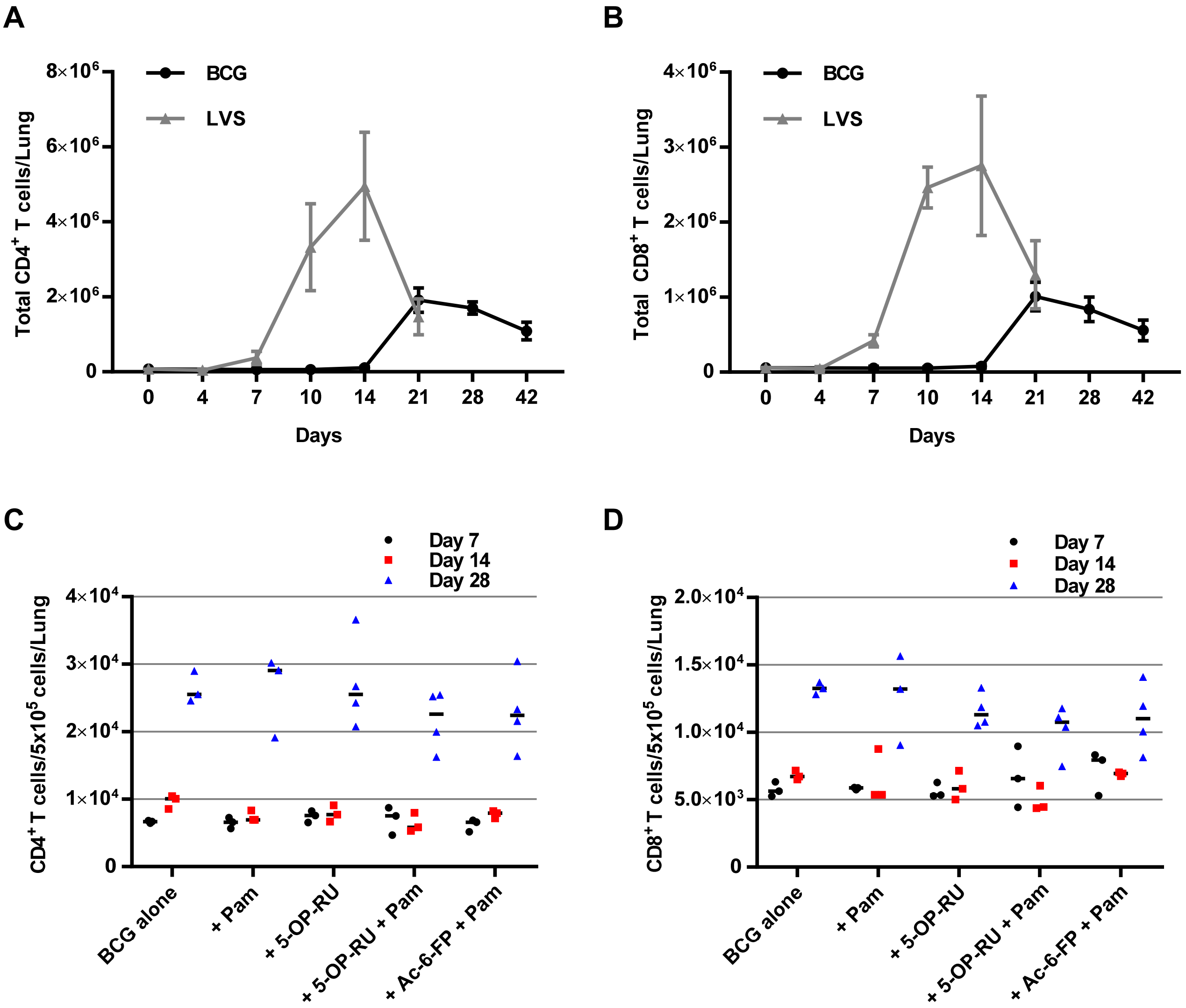

Supplementary Figure 1. CD4$^+$ and CD8$^+$ T cell numbers in the lungs during BCG and LVS pulmonary infections. Enumeration of the total number of CD4$^+$ T cells (**A**) CD8$^+$ T cells (**B**) in the lungs of WT mice infected IN with 10^6 BCG CFU and 2×10^2 LVS CFU. Data show the mean ± SEM (n= 3-4 mice). In (**C**) and (**D**), mice were intranasally administered BCG on day 0, MAIT cell ligand 5-OP-RU (or Ac-6-FP as negative control) + Pam on day 1, and 5-OP-RU or Ac-6-FP alone on days 2 and 3. Enumeration of the number of CD4$^+$ T cells (**C**) CD8$^+$ T cells (**D**) in the lungs of mice on days 7, 14, and 28 after the treatments. Data show individual values and the median (n = 3-4 mice). All data are representative of three independent experiments.

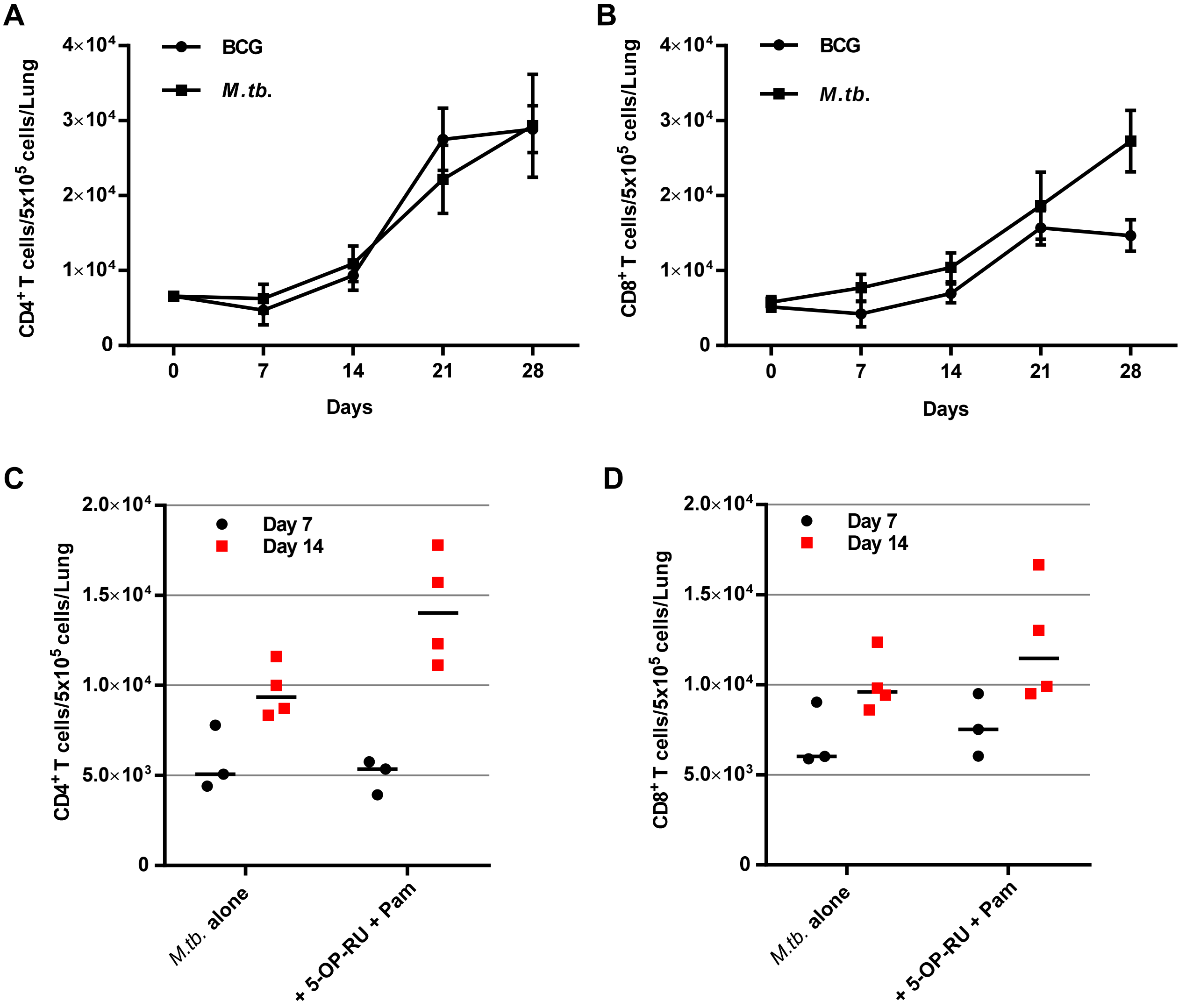

Supplementary Figure 2. CD4⁺ and CD8⁺ T cell numbers in the lungs during BCG and *M. tb.* pulmonary infections and following TLR2/6 agonist plus 5-OP-RU treatment. Enumeration of the number of $CD4^+$ T cells (**A**) $CD8^+$ T cells (**B**) in WT mice infected IN with 10^6 BCG CFU or 1×10^2 *M. tb*. via aerosol. Data show the mean ± SEM (n = 3-4 mice). In (**C**) and (**D**), mice were administered *M. tb*. via aerosol on day 0, MAIT cell ligand 5-OP-RU (or Ac-6-FP as negative control) + Pam on day 1, and 5-OP-RU or Ac-6-FP alone on days 2 and 3. Enumeration of the number of $CD4^+$ T cells (**C**) $CD8^+$ T cells (**D**) in the lungs of mice on days 7 and 14 after the treatments. Data show individual values and the median (n = 3-4 mice)**.** All data are representative of three independent experiments.
